# Monocyte expression of MICA enhances the Natural Killer Cell response to antibody-coated tumor targets

**DOI:** 10.1186/2051-1426-2-S3-P252

**Published:** 2014-11-06

**Authors:** Amanda Harper, Neela Bhave, Kallan Opheim, Eric Leudke, Robin Parihar, Prashant Trikha, William Carson

**Affiliations:** 1 Comprehensive Cancer Center, The Ohio State University, Columbus, OH, USA; 2 Department of Surgery, The Ohio State University, Columbus, OH, USA; 3 Department of Pediatrics, The Cleveland Clinic, Cleveland, OH, USA

## 

Natural Killer (NK) cells are large granular lymphocytes that are uniquely equipped to promote the anti-tumor response via communication with other immune cell types in the tumor microenvironment. We have shown that NK cells secrete a distinct profile of immune stimulatory factors e.g., interferon-gamma (IFN-γ) with potent anti-tumor activity in response to dual stimulation with antibody (Ab)-coated tumor cells and cytokines, such as interleukin (IL)-12 and that this response is significantly enhanced 10 fold in the presence of autologous monocytes (p < 0.05). Similar results were obtained when NK cell secretion of TNF-alpha (a) and MIP1-a were examined. This enhancement of the NK cell cytokine response by monocytes held true for every known stimulator of NK cell IFN-γ production. Further, autologous monocytes significantly increased the ability of IL-12 activated NK cells to lyse Trastuzumab-coated breast cancer cells in an ADCC assay (p < 0.05). Monocyte enhancement of NK cell activity was shown to be dependent on direct cell-cell contact as determined by a transwell assay. We hypothesized that NK cell effector functions against Ab-coated tumor cells were enhanced via binding of the stimulatory ligand MICA on monocytes to the NKG2D receptor on NK cells. Activation of monocytes with bacterial components (e.g., LPS) or IFN-a led to increased surface expression of the NKG2D ligand MICA and further enhanced the ability of monocytes to act as stimulators of NK cell cytokine secretion. The stimulatory effects of MICA-positive monocytes were duplicated via use of a MICA over-expressing cell line (C1R-MICA) (inducing a 4 fold increase in IFN-γ production) but not the parental MICA-negative cell line. Down-modulation of MICA via siRNA or incubation with a MICA neutralizing FAb fragment prior to co-culture of monocytes with NK cells led to a significant reduction in NK cell IFN-γ secretion (p < 0.05). Blockade of NKG2D on NK cells with a neutralizing Ab also reduced IFN-γ production in this co-culture system. *In vivo*, depletion of monocytes from Balb/c mice resulted in decreased IFN-γ production by murine NK cells upon exposure to Trastuzumab-coated tumor cells and IL-12. Extended depletion of monocytes *in vivo *abrogated the immune response to combination therapy with Trastuzumab and IL-12, resulting in significantly larger tumors in monocyte-depleted mice in comparison to mock-depleted controls (p < 0.02). These data suggest that direct cell-cell interaction between NK cells and monocytes may constitute an important mechanism for enhancement of the NK cell anti-tumor response in the setting of monoclonal Ab therapy for cancer.

